# Simulating epileptic seizures using the bidomain model

**DOI:** 10.1038/s41598-022-12101-y

**Published:** 2022-06-16

**Authors:** Jakob Schreiner, Kent-Andre Mardal

**Affiliations:** 1 Simula Research Laboratory, Oslo, 0164 Norway; 2 Expert Analytics AS, Oslo, 0179 Norway; 3 Department of Mathematics, University of Oslo, Oslo, 0851 Norway

**Keywords:** Applied mathematics, Epilepsy

## Abstract

Epileptic seizures are due to excessive and synchronous neural activity. Extensive modelling of seizures has been done on the neuronal level, but it remains a challenge to scale these models up to whole brain models. Measurements of the brain’s activity over several spatiotemporal scales follow a power-law distribution in terms of frequency. During normal brain activity, the power-law exponent is often found to be around 2 for frequencies between a few Hz and up to 150 Hz, but is higher during seizures and for higher frequencies. The Bidomain model has been used with success in modelling the electrical activity of the heart, but has been explored far less in the context of the brain. This study extends previous models of epileptic seizures on the neuronal level to the whole brain using the Bidomain model. Our approach is evaluated in terms of power-law distributions. The electric potentials were simulated in 7 idealized two-dimensional models and 3 three-dimensional patient-specific models derived from magnetic resonance images (MRI). Computed electric potentials were found to follow power-law distributions with slopes ranging from 2 to 5 for frequencies greater than 10–30 Hz.

## Introduction

Epilepsy is the fourth largest neurological disorder with a prevalence of 7.1 per thousand in all ages^1^. The condition involves seizures of abnormal brain activity, where waves of electrical activity propagate through the brain and cause a variety of symptoms, ranging from absent-mindedness to loss of consciousness. An epileptic seizure is due to excessive or synchronous neural activity in the brain^2,3^. Extensive modeling has been performed on the neuronal level, but only few works^4,5^, have to the authors’ knowledge, scaled up the neuronal modelling to brain-wide simulations. The so-called Bidomain model, widely used for cardiac modeling, allows for this upscaling. Here, we investigate the simulation of seizures based on a specific neuronal model by Cressman et al.^6,7^ developed for epileptic seizures which is then combined with the Bidomain model.

To compare the computational model with actual seizures we have here chosen to quantify the simulation results in terms of the power-law distribution of the power spectral density measured at specific points, as the spectrum can be easily compared with electroencephalograms (EEGs) and the power-law allows for quantification across several scales. Specifically, we will consider a power-law distribution of the form $p \sim f^{-\beta }$, where *f* is the frequency. Measurements of brain activity exhibit a $f^{-\beta }$-like power spectrum at many spatiotemporal scales, see He^8^ for a review. During normal brain activity, the value of $\beta$ is often found to be around 2^9–14^ for frequencies in the range 1–150 Hz, while the exponent is higher for higher frequencies^15^. Values between 0.5 and 3 have been explained in a theoretical and computational study^16^ and has been shown to vary with spatial distribution in quasi-static models^17^. In contrast, the neuronal model for seizures^6,7^ that is being considered here, displays power-law behaviour, with $\beta \approx 3.5$ but only in the range $f > 100$ Hz. Furthermore, Schreiner et al.^18^ have recently demonstrated that the power-law under ECT induced seizure, as measured with EEG, is in the range 3.4–4.2.

In this paper, we will investigate whether the Bidomain model can be combined with a neuronal model for epileptic seizures, together with MRI-based geometries and diffusion parameters of the whole brain, to yield a plausible power-law distribution. The Bidomain model^19–23^, which is widely used in cardiac modeling, addresses the interaction of the intra-cellular and extra-cellular compartments by homogenization, such that every point in the model represent a volume average of both intra- and extra-cellular compartments. A main difference between the brain and the heart is however that the cells of the heart are very structured and that each cell is locally connected to only neighbouring cells. This structure is not present in the brain and only the extra-cellular compartment of the brain is locally connected. However, under seizures, the extra-cellular compartment play a significant role and as such the Bidomain model might be able to capture the complex waves that arise.

The Bidomain model has previously been used to simulate neuronal resting-state potentials during transcranial direct current stimulation^5^ and an ischemic region in a post-stroke patient^24,25^. The Bidomain model includes the extracellular and the transmembrane potential, and couples naturally with dynamical models for the transmembrane potential. Bai et al.^26^ simulated the electric field following direct current stimulation, representing the head as a passive volume conductor, but incorporating a Hodgkin-Huxley^27^ type model in the cortical tissue. Their model captures neuronal activation, but not neuronal communication. There are other well-established models based on Poisson’s equation describing electric currents in the different tissues in the brain, see e.g. Næss et al.^28^ or Lee et al.^29^. However, these models do not capture the temporal evolution of the electric field. Alternative data-driven models have been developed for seizure dynamics, such as by Jirsa et al.^30^, and extended to the whole brain using network models based on brain functional imaging^31^.

Our task in this paper is to investigate whether macroscopic physics-based models, formulated as partial differential equations, such as the Bidomain model, combined with neuronal models for epileptic seizures give rise to plausible power spectra. Using the Bidomain model, we simulate the electric potential in the brain in an idealised two-dimensional (2D) geometry and an MRI-based three-dimensional (3D) geometry. Specifically, we utilize the neuronal model by Cressman et al.^6,7^ in which the excitability of the neurons is parametrized in terms of extracellular potassium in order to differentiate between seizures and normal activity. We remark that the Cressman model targets individual neurons. As such, one aim of this paper is to investigate to what extent the Bidomain equations together with a neural seizure model such Cressman’s give rise to frequencies in electric potential as measured by EEG on the outside of the skull.

We will consider the importance of the geometry, the cell-model, and the white matter (WM) anisotropy. To assess our approach we compare the power spectrum of the computed electric potential with the power-law behaviour of the EEG power spectrum.

## Methods

### Cell models

**Figure 1:**
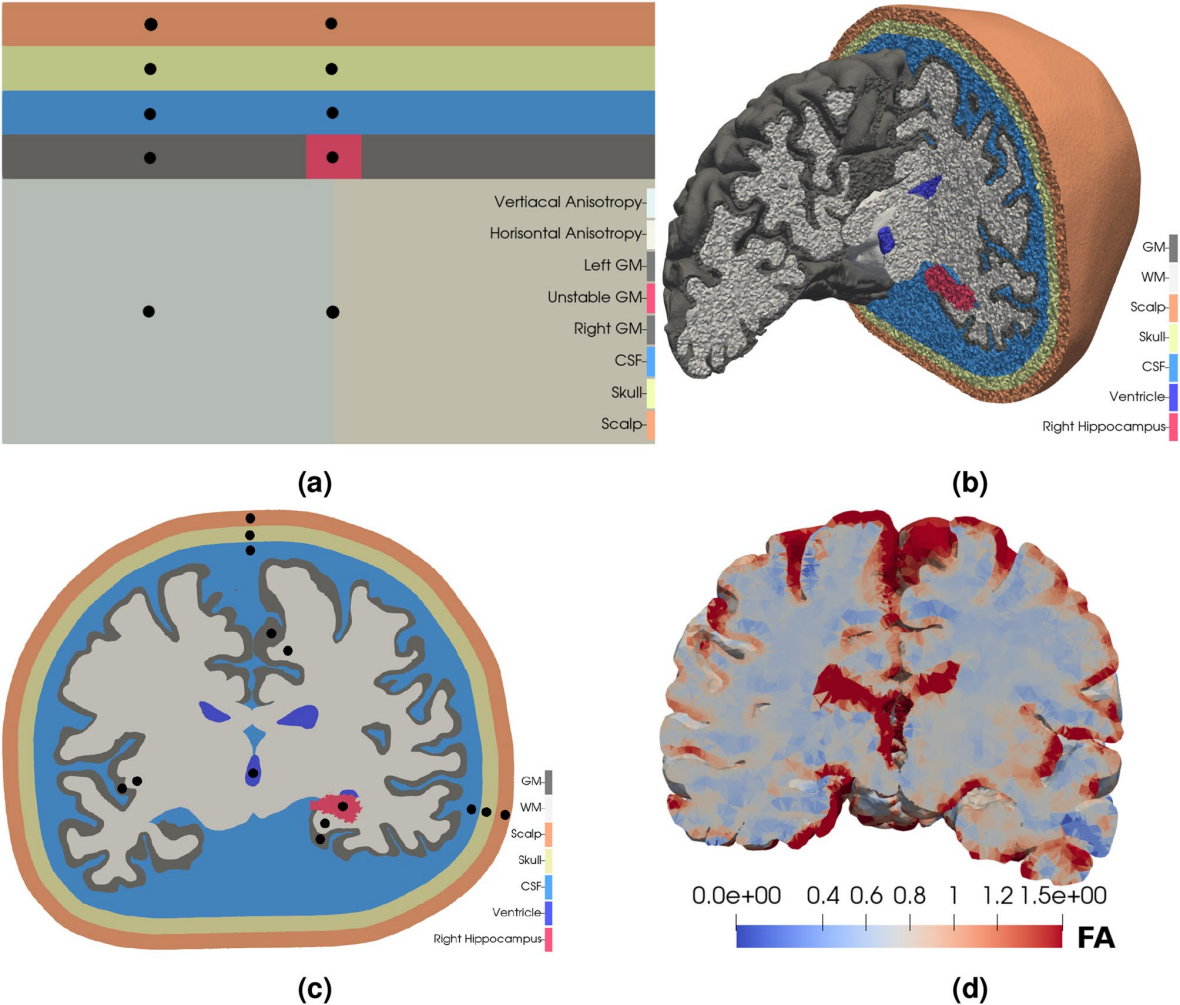
Computational geometries (**a**) An idealised two-dimensional model of a slice of the head with, from the top, scalp (orange), skull (yellow/pale green), cerebrospinal fluid (blue), grey matter (dark grey and red), and white matter (two shades of light grey). The central grey matter (red) is modelled by an unstable neuronal model, Model B, while the rest of the grey matter (dark grey) is modelled by a stable neuronal model, model A. The conductivity is isotropic, except in the white matter. The anisotropic conductivity tensor in the white matter is oriented vertically in the left part and horizontally in the right part. The simulated electric potential is measured at locations marked by black dots (**b**) The three-dimensional patient specific head model. The colours are the same as in (**a**), however, the ventricles are marked dark blue, and the right hippocampus is marked in red. Note that the ventricles are modelled the same way as the cerebrospinal fluid. As in (**a**), the grey matter is modelled by Model A, while the right hippocampus is modelled by Model B. (**c**) A slice of the head model in (**b**). The simulated electric potential is measured at locations marked by the black dots. (**d**) The mean diffusivity is highest in the ventricles and the cerebrospinal fluid, both showing up in red. The mean diffusivity is $1/3~Tr{(M)}$, where $M$ is the conductivity tensor. The conductivities are measured in $\text {mS}/\text {cm}$.

**Figure 2:**
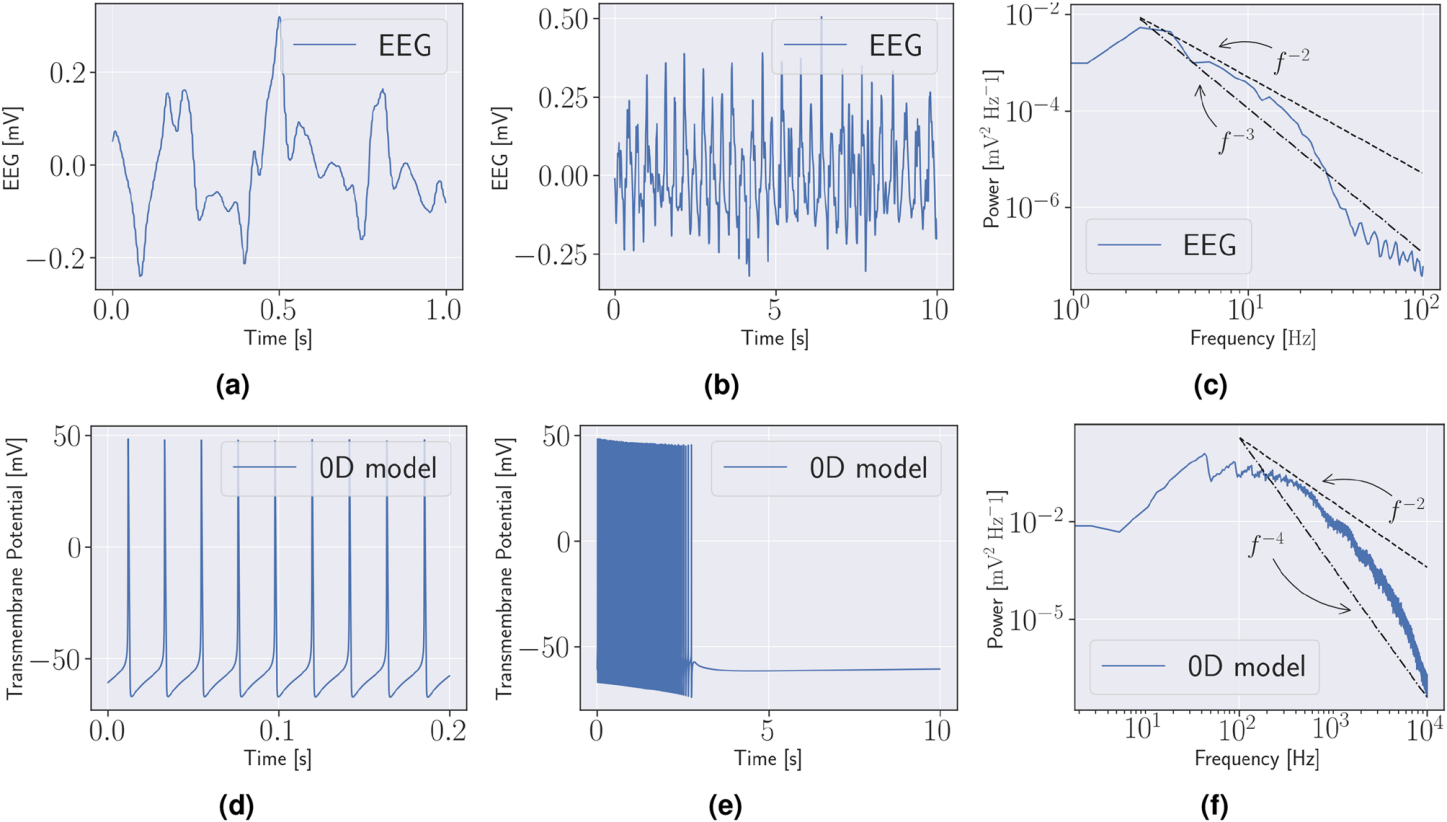
EEG and cell model The top row show an electroencephalogram (EEG) from a patient experiencing a seizure. (**a**) Shows 1 s of the EEG, (**b**) shows 10 s, and (**c**) the power spectrum computed from the 10 s segment in (**b**). The bottom row shows the simulated transmembrane potential from model B. (**d**) Shows 0.2 s, (**e**) 10 s with an initial period of spikes, a burst, followed by a period of quiescence, (**f**) the power spectrum computed from the burst in (**e**). The straight lines illustrate power-laws of *f* with slopes − 2 to − 4.

We considered two different types of geometries: (1) an idealised 2D geometry Fig. 1a, and (2), a patient-specific 3D geometry, Fig. 1b,c based on T1-weighted and diffusion weighted MR images, with mean diffusivity illustrated in Fig. 1d. On these geometries the Bidomain model is coupled with neuronal models which are parametrized to either a seizure-like (unstable) or normal state (stable). An overview of the different models is provided in Table 1. Both model A and B are neuronal cell models introduced in Cressman et al.^6,7^, but Model B has high steady state extracellular potassium (K^+^) concentration, ${{\,\mathrm{k_{o, \infty }}\,}}$ = 8 mM, compared to 4 mM in Model A. As such, Model B is in a seizure-like state, and alternates between periods of spike trains (bursts) and quiescence, seen in Fig. 2. We remark that there is a significant discrepancy in frequency content between the EEG, i.e. Fig. 2a–c and Cressman model for individual neurons, see Fig. 2d–f. We compared models by counting the number of spikes, as also done in Erhardt et al.^4^, as well as computing the power-spectra.

**Table 1:** A short summary of the models considered in this study.

Model	Spatial dimension	Mesh	White matter cell model	Anisotropy
**Ordinary differential equations**				
Model A	0	–	Cressman (stable)	–
Model B	0	–	Cressman (unstable)	–
**Ordinary and partial differential equations**				
Model C	2	Square	–	Yes
Model D	2	Square	Cressman (stable)	Yes
Model E	3	Head	–	No
Model F	3	Head	–	Yes
Model G	3	Head	Cressman (stable)	No

Models A and B are systems of ordinary differential equations (ODEs) describing cell membrane dynamics, and are coupled with the Bidomain model, a partial differential equations (PDE) in the two-dimensional models, C and D, as well as the three-dimensional models, E, F, and G.

### Two-dimensional models

Models C and D represent idealized 2D geometries, as shown in Fig. 1a. The geometry is an idealisation of a cortical slice and measures 60 mm × 40 mm. The grey matter (GM), cerebrospinal fluid (CSF), skull, and scalp each measures 4 mm across. We used this idealisation to study the effect of the tissue surrounding the brain, as well as the effect of an anisotropic conductivity tensor on the interaction between the Cressman model and the Bidomain model. The GM (dark grey) was modelled by the stable Cressmann model, Model A, except for the central red square in Fig. 1a, which was modelled by the unstable Cressmann model, Model B. The WM (light grey) had an anisotropic conductivity tensor while the conductivity in the GM, CSF, skull and scalp was isotropic. We considered two variations of this model, one with no cell model in the WM, termed Model C, and one with Model A in the WM, termed Model D. In the 2D models, the GM and WM was further distinguished by the isotropic conductivity in the GM and anisotropic conductivity in the WM. The anisotropic conductivity tensor in the WM of the 2D models was

1 $$\begin{aligned} \begin{pmatrix} \sigma _t &{} 0 \\ 0 &{} \sigma _l \end{pmatrix}, \end{aligned}$$

where $\sigma _l = 10\sigma _t$ and $\sigma _t = 0.1$, that is, the principal component of the conductivity tensor was oriented vertically. In the right part of the WM, $\sigma _l$ and $\sigma _t$ in Eq. (1) are swapped, i.e., the conductivity tensor is oriented horizontally.

### Patient-specific three-dimensional models

The three patient-specific 3D models (Models E, F, G) were based on surfaces of the pia, WM, and ventricles. The surfaces were reconstructed from a MRI of a human head using FreeSurfer^32,33^. We represented the subarachnoid space (SAS) by the convex hull of the pia expanded 3mm along the outward facing normal vector. The skull and scalp were similarly defined as the expanded convex hull of the SAS and skull respectively. The resulting mesh is shown in Fig. 1b, and has 72 million cells. Volume meshes were created with SVMTK^34^, which is based on CGAL^35^. As in the 2D models, the GM is modelled by Model A, except for the right hippocampus, which is modelled by model B. Model E has isotropic WM conductivity and no WM cell model. Model F has an anisotropic conductivity tensor in the WM, and no WM cell model. The conductivity tensor was assumed proportional to the water diffusion tensor^36,37^, which was constructed from the diffusion tensor imaging (DTI) using FreeSurfer. That is, we have followed Tuch et al. in quantitatively estimating the anisotropic conductivity tensor from water diffusion in the brain^36^, an approach validated in the study by Bangera et al.^37^. The approach used by Bangera et al. assumes that the scaling between the diffusion tensor and the conductivity tensor is the same throughout the brain. The mean diffusivity of the conductivity tensor is shown in Fig. 1d. Model G has isotropic WM conductivity and Model A as a WM cell model. Experiments with model G were also run with a Morris-Lecar model^38^ in the WM, rather than Model A. The small black circles in Fig. 1a,c show locations where time traces of the potential were sampled. The power spectral density (PSD) of the electric potential were estimated using Welch’s method^39^ implemented in *scipy* version 1.6.1^40^.

The approval for MRI-observations was retrieved by the Regional Committee for Medical and Health Research Ethics (REK) of Health Region South-East, Norway (2018/1093). The study participants were included after written and oral informed consent. All methods were performed in accordance with the relevant guidelines and regulations.

### The Bidomain model

Following^23^, the Bidomain model reads

2 $$\begin{aligned} \begin{aligned} \nabla \cdot (M_i\nabla v) + \nabla \cdot (M_i \nabla u_e)&= \chi C_m \frac{\partial v}{\partial t} + \chi I_{ion} \\ \nabla \cdot (M_i\nabla v) + \nabla \cdot ((M_i + M_e)\nabla u_e)&= 0, \end{aligned} \end{aligned}$$

with the boundary conditions

3 $$\begin{aligned} \begin{aligned} (M_i\nabla v + M_i\nabla u_e) \cdot \mathbf {n}&= 0 \\ (M_e \nabla u_e) \cdot \mathbf {n}&= 0. \end{aligned} \end{aligned}$$

Here, $v$ and $u_e$ are the transmembrane and extracellular potential respectively, and $M_i$ and $M_e$ are the intracellular and extracellular conductivity respectively. Furthermore, $C_m$ is the cell membrane capacitance and $\chi$ is the membrane surface area per unit volume. The ionic current across the cell membrane, $I_{ion}$, is modelled by the Cressmann model. In the CSF, skull and scalp $M_i$ is 4 orders of magnitude less than $M_e$ in the GM and WM. The ventricles are modelled the same way as the CSF. Table 2 provides an overview of physical parameters used in this study. Values for $C_m$, $\chi$, and GM and GM conductivities are taken from Dougherty et al.^5^, while CSF, skull and scalp conductivities are taken from Vorwerk et al.^41^.

The Bidomain model coupled with the Cressman model was solved with a second order splitting scheme in time, see^23^ for a derivation, and a time step of 0.025 ms. The ordinary differential equations were solved only in the GM unless specified otherwise, using the forward Euler method. In space, Equation 2 was discretised with the finite element method using first order continuous Lagrange elements. The linear system was solved using the generalised minimal residual method (GMRES) preconditioned with an algebraic multigrid method with relative tolerance set to $10^{-5}$ and absolute tolerance set to $10^{-50}$. The software used to solve the equations is based on *cbcbeat*^42^ built around the FEniCS project version 2019.1.0^43,44^.

**Table 2:** Physical parameters.

	Value	Units
Membrane capacitance ($C_m$)	1	*μF*
Cell membrane surface per unit volume ($\chi$)	$1.26 \times 10^3$	$1/\text {cm}$

Tissue type	Conductivity Intracellular	$\textbf {mS}/\textbf {cm}$ Extracellular
Grey matter	1	2.78
White matter	1	1.26
Cerebrospinal fluid	$10^{-4}$	17
Skull	$10^{-4}$	0.1
Skin	$10^{-4}$	4.3

## Results

### Cell models

The stable Cressman model, Model A, spiked 5 times, then remained quiet, and did not exhibit any bursts. In contrast, during the 10 s simulation the unstable Cressman model, Model B, spiked 241 times in 5.7s, then entered a period of quiescence, see Fig. 2e. It continues to alternate between bursts and periods of quiescence. This is similar to what was observed in the study by Erhardt et al.^4^.

### Two-dimensional models

**Figure 3:**
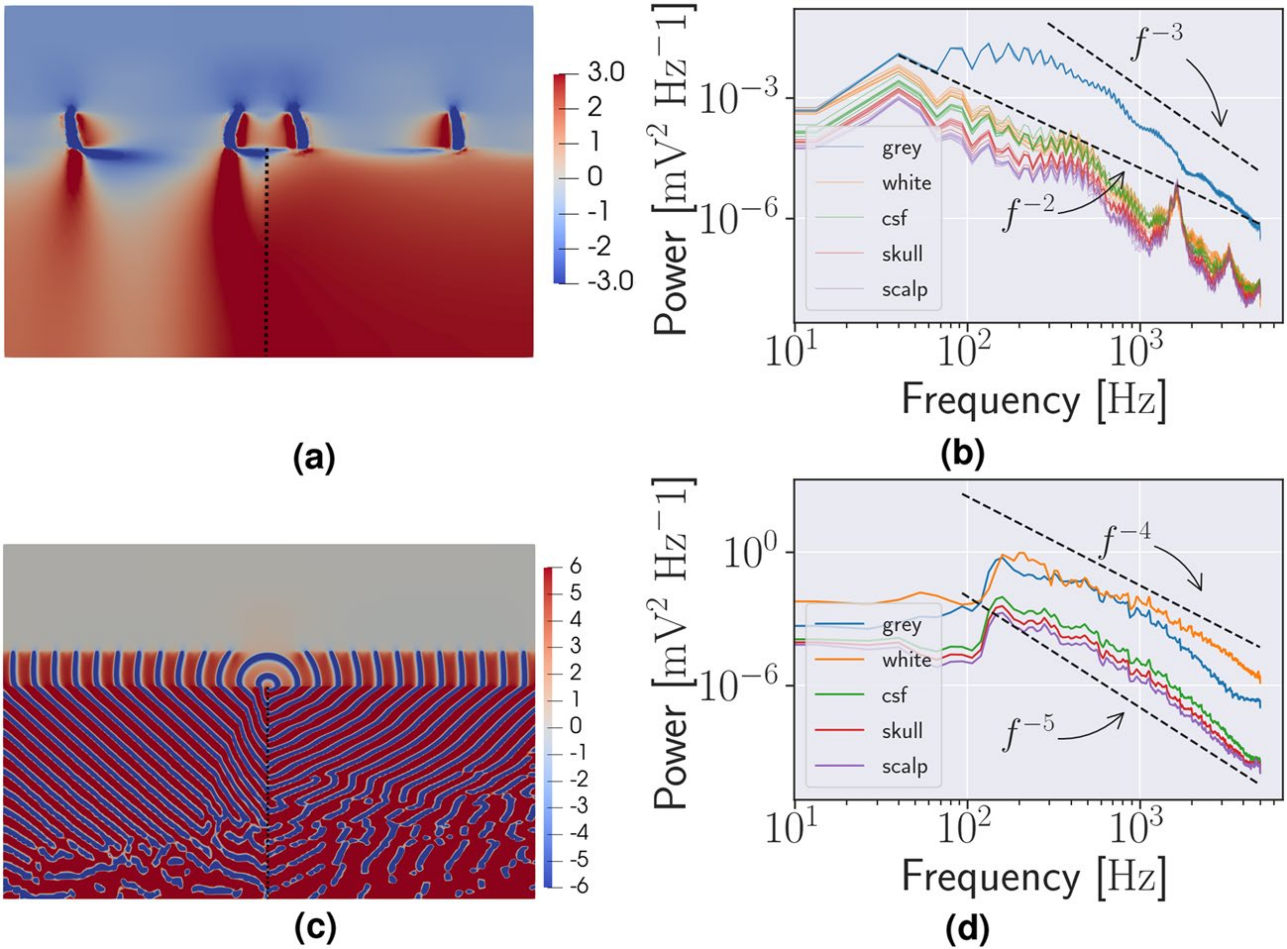
Two-dimensional simulations and power spectra (**a**) A snapshot of the simulated extracellular potential in Model C. The waves spread through the grey matter from the central unstable region. The unstable region is marked in Fig. 1a. (**b**) The extracellular potential in the central (unstable) grey matter. Variations to the thickness of the cerebrospinal fluid (CSF), skull and scalp as well as the individual components of the conductivity tensor by a factor of 1/3 affect the magnitude of the power spectral density (PSD) in the white matter, CSF, skull and scalp but not the grey matter. Each variation is represented by a thin line. (**c**) A snapshot of the simulated extracellular potential in Model D. Waves spread from the GM from the unstable central region, and also into the white matter. (**d**) The PSD of the extracellular potential in the different tissues with no cell model in the WM.

In the idealized 2D, waves originate in the central unstable region and spread along the GM in both the model with and without a WM cell model, Model C and Model D, respectively, see Fig. 3a,c. To count the spikes, the potential was measured in the locations indicated in Fig. 1a and adjusted accordingly as the thickness of the CSF, skull, and scalp was changed. There were 135 identifiable spikes in the initial burst in the Model C lasting 3.1 s, as such the burst duration is shorter in Model C, but the number of spikes per second is roughly similar to Model B. Model D spiked continuously during the 10 s simulation, with no identifiable bursts, counting 1873 spikes. Furthermore, the magnitude of the spikes were approximately doubled and the spike frequency was increased by a factor 4.3 compared to Model C. As such, it appears that the coupling represented by the Bidomain model may both accelerate and decelerate the neuronal firing, depending on the WM model. That is, in Fig. 3b there is a significant different in the slope of the grey mater and white matter and the other regions appear to mirror the white matter. In the case of a WM Cressman model, Fig. 3d, the slope appears similar in all regions. Finally, the Bidomain model enable spreading from an unstable domain to the complete domain, even though the bulk of the domain is initialized by stable parameters of the Cressman model, i.e. extracellular potassium of 4 mM.

#### Spatial resolution

The effect of the mesh resolution was investigated in detail in 2D. The number of spikes during the 10 s simulation of Model C is listed in Table 3 and demonstrate that a mesh with 1738 cells adequately resolves the travelling waves, see Fig. 3a. For coarser meshes the waves failed to propagate. The 1738 mesh mesh had 5 cells across the GM. The resolution was higher in the GM than in the CSF. In the simulations with a cell model in the WM, the resolution in the GM and WM was the same.

**Table 3:** The number of spikes and the number of cells in two-dimensional simulations in the domain in Fig. 1a.

# Cells	# Spikes
6120	135
1738	135
514	132 (3)

The spikes were counted throughout the grey matter. Only in the coarsest case did any waves fail to propagate from the unstable region. In that case, only the first three (i parenthesis) spread to the stable parts of the grey matter. The total number of spikes were counted over a 10 s interval.

#### The effect of anisotropy in two- and three-dimensional models

The effect of the anisotropic conductivity tensor in Model C was evident in the magnitude of the extracellular potential. That is, the passing waves in the GM propagate further into the WM see Fig. 3a. In detail, in the left part, where the conductivity tensor is oriented vertically, the extracellular potential is affected along the complete WM column of 24 mm. In the right part, where the conductivity tensor is oriented horizontally, the corresponding propagation is only 2-3 mm. In Model D, the 2D model with a WM cell model, the effect of the anisotropic conductivity tensor is evident in the deflection of the waves in the WM, as seen in Fig. 3c. In both Model C and D, the orientation of the conductivity tensor affects the magnitude of the extracellular potential, and as such, the magnitude of the PSD, but not the power-law exponent.

#### The effect of cerebrospinal fluid skull and scalp

To investigate the sensitivity of the thickness of the CSF, skull, and scalp on Model C, we performed two new simulations increasing and decreasing the thickness of the CSF, skull, and scalp by a factor of 1/3. Similarly, investigating the sensitivity to the anisotropic conductivity tensor, we ran four new experiments independently increasing and decreasing $\sigma _l$ and $\sigma _t$ by a factor of 1/3. As such, the variation in CSF, skull, and scalp thickness and anisotropy accounts for 7 different simulations. The variations to the thickness of the CSF, skull, and scalp as well as the individual components of the conductivity tensor resulted in minor variations in the magnitude of the PSD, seen in Fig. 3b. Here, notice that for each colour there are 7 lines representing the different simulations. We also remark that the changes in the potential due to these variations are smaller than the differences in the potential between the compartment (WM, CSF, skull, scalp). The PSD in the GM was unaffected by these perturbations.

### Patient specific models

**Figure 4:**
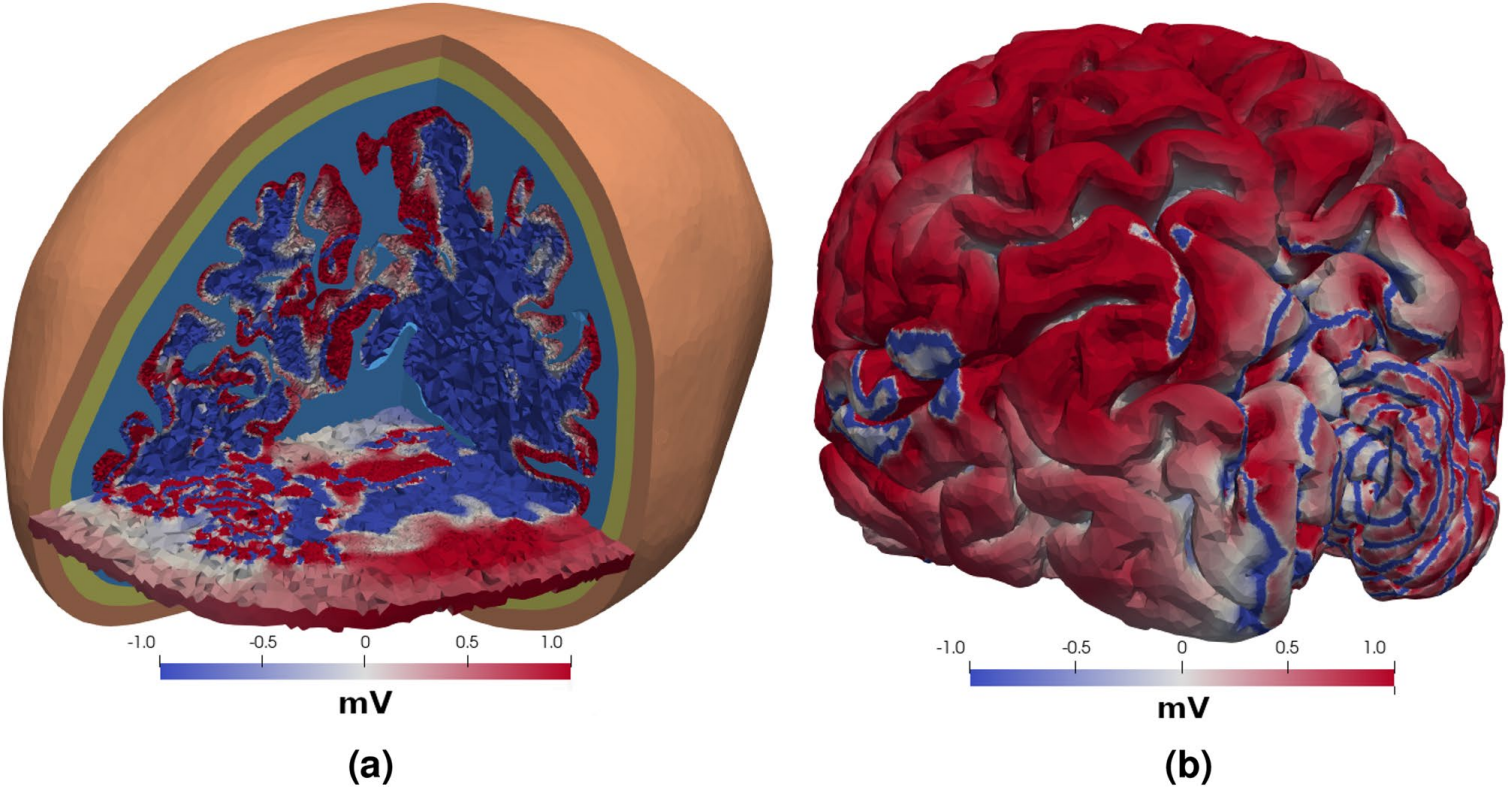
Three-dimensional simulations Two visualisations of the extracellular potential in the head and on the brain surface after 100 ms. The colormap is scaled to highlight the waves, and does not cover the range of the extracellular potential. (**a**) The extracellular potential throughout the brain and the surrounding tissue in a corner of the head. The surrounding tissue is shown in colors similar to Fig. 1b, while the magnitude of the extracelular potential shown is shown in the grey matter and white matter. The horizontal slice also shown the magnitude of the extracelular potential in the cerebrospinal fluid, skull and scalp. (**b**) The extracelular potential on the surface of the brain.

**Figure 5:**
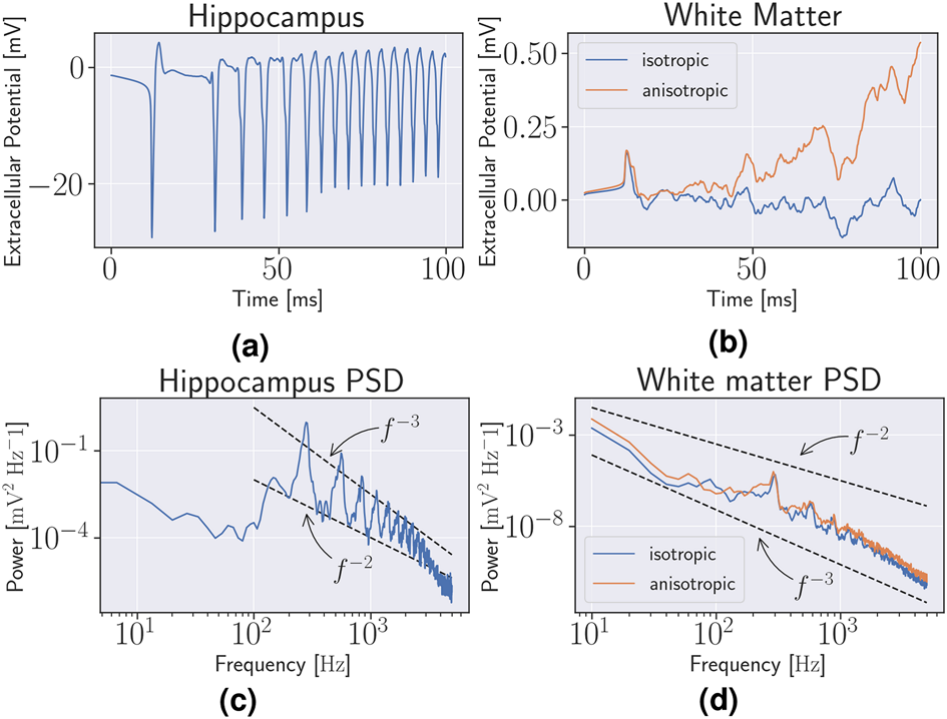
Three-dimensional spikes and power spectra (**a**) The simulated extracellular potential in the right hippocampus in Model E. (**b**) The extracellular potential in the white matter in the left hemisphere from Model E and Model F. (**c**) The power spectrum of the potential in (**a**). (**c**) The power spectrum of the potential in (**b**).

The geometry of the 3D model is shown in Fig. 1b and the measurement locations are shown in Fig. 1c. The mesh contained 72 million finite element cells and the resolution was similar to the idealized, resolved 2D geometry in the sense that there were 5 cells across the GM. The individual cells can be seen in the slices. In 3D, waves spread from the destabilised hippocampus, and through the surrounding GM in the model with an isotropic conductivity tensor, Model E, and the model with an anisotropic conductivity tensor, as seen in Fig. 4 after 100 ms. In the hippocampus, the extracellular voltage traces were similar to Model B. There were between 10 and 13 spikes in the hippocampus during the 100 ms of the simulation of model E and F, see Fig. 5a, depending on the exact measurement location. That is, the number of spikes per second is between 100 and 130, which is more than twice as high as in the idealized 2D model without WM model, Model C. The anisotropic conductivity tensor in Model F did not affect the frequencies in the WM or the GM, but the magnitude of the extracellular potential was greater throughout the brain, as seen in Fig. 5b. In Model G, the model with a stable Cressman model in WM, there were no spiking in the hippocampus. With a Morris-Lecar model^38^, the number of spikes were the same as models E and F.

### Power-laws in two dimensions

The $\beta$-parameter of the power-law of the power spectrum of Model B, see Fig. 2f, was between 2 and 3 for $f$ between 100 and 5000 Hz. The PSD of the extracellular potential of Model C followed a power-law with $\beta \approx 2$ for $f$ between 40 Hz and 5000 Hz in the WM, and $\beta \approx 3$ for $f$ between 300 Hz and 5000 Hz in the GM, see Fig. 3b. The PSD of the extracellular potential in Model D followed a power-law with an exponent close to 5 in the “passive” tissue, the skull, scalp, and CSF, and 4 in the “active” tissue, the GM and WM. Both power-laws are for $f$ between 150 Hz and 5000 Hz, see Fig. 3d.

### Power-laws in three dimensions

The power-spectra of Model E and Model F were identical in the hippocampus, and is shown in Fig. 5c. The peak of the PSD was at 300 Hz. The two black lines show a power-law corresponding to $\beta = 2$ and $\beta = 3$ for $f$ between 100 and 5000 Hz. The power-law in the WM in the model with an isotropic conductivity tensor, Model E, and anisotropic conductivity tensor, Model F, is shown in Fig. 5d, with a peak located at 10 Hz. The two black lines illustrate power-laws with slopes $\beta = 3$ and $\beta = 2$ for $f$ between 10 and 5000 Hz. In general, we see that the Bidomain model when combined with Cressman yields a power-spectra mode similar to that of EEG both in 2D, Fig. 3b,d and 3D, Fig. 5d,c than the stand-alone Cressman model, Fig 2f.

## Discussion

In this paper we simulated the electrical potential in a human brain during a seizure using the Bidomain model with a Cressman^6,7^ excitation model in 10 geometries (7 idealized 2D models and 3 MR based three-dimensional models). The Bidomain-Cressman model resulted in electrical waves propagating through the brain from unstable to stable regions as also observed in Erhardt et al.^4^. The PSD of these waves follow a power-law distribution similar to what is found in EEGs. That is, the power-law slopes during a seizure have values from 2 to 5, depending on the model. In detail, for all models that did not have an active model in WM the slope was found to be between 2 and 3, while for the idealized model with a stable Cressman model in the WM a slope of between 4 and 5 was observed. The values are in agreement with previous studies^9–15,45–47^, where values between 2 and 4 are reported, although none of these studies investigate seizures in humans. Observed power-law slope in EEG was recently reported to be between 3.4 and 4.2^18^ during ECT-induced seizures. We remark that it is interesting to observe that the Bidomain model extended the slope of the power-law in the Cressman model from 100 to 5000 Hz down to around 30 Hz. In EEGs, frequencies from a few Hz to a few hundred Hz are commonly observed, and as such the Bidomain model brings the Cressman models into the regime of interesting frequencies.

The anisotropic conductivity tensor did not have a significant effect on the distribution of power in the PSD in any of the models, as compared with the isotropic conductivity. However, the anisotropy has a significant effect on the magnitude of the extracellular potential. In this study we have used physiologically realistic values from the literature^5^, although the context was here isotropic. Previous studies have found that tissue anisotropy affects electric field magnitudes^29,37^, but we are unaware of studies investigating the effect on frequency content.

We have investigated the effect of a WM cell model in both 2D and three-dimensional simulations. While using the stable Cressman model in the WM had a significant effect on burst duration, power-law slope and frequency range in 2D, the corresponding simulation in three-dimensional demonstrated that the unstable hippocampus was prevented by the WM from spiking. A further experiment with a Morris-Lecar model^38^ in the WM in a three-dimensional simulation had negligible effect in the simulated potentials when compared with no WM cell model. A previous study of the coupling of the Cressman and Bidomain models showed that there is a balance between the conductivity and the size of the unstable region, i.e., the right hippocampus^4^. If the conductivity is too high, or the unstable region too small, the stable regions of the model dominates and spiking stops. This issue has not been addressed in the three-dimensional models due to the computational costs. Compared to the  100 billion neurons and equal number of glia in the brain^48^, our realistic head models consist of 70 million cells. The three-dimensional simulations of 100 ms of the brain activity took 200 h with 16 cores using 16 GB of memory each.

### Limitations

The Bidomain model is widely used for cardiac applications, and its application to the brain is uncertain. In particular, the intra-cellular compartment is much more complex in the brain. In seizures, however, the extra-cellular compartment plays a major role and as such the Bidomain model may be a promising candidate model. Further, the Bidomain model assumes a purely resistive medium^23^, and the possibly frequency dependent properties of the extracellular medium may limit the applicability of the Bidomain model. According to Nunez and Srinivasan, typical EEG frequencies less than 100 Hz are not affected by capacitative effects of the extracellular medium^49^. Furthermore, the resistive properties of the extracellular medium have been reported to be independent of the frequency^50^ or weakly frequency dependent^51^. More recent studies, however, suggest that there may be important frequency dependencies of the extracellular medium. Gomes et al. found a strong frequency dependence in the extracellular conductivity^52^, linking the frequency dependence to ionic diffusion. A possible explanation of the discrepancies lies in the measurement technique, and the findings are controversial, see the discussion^53,54^ following the study by Gomes et al.^52^. Assuming ionic diffusion is the origin of the frequency dependence in the extracellular conductivity, Bédard and Destexhe show that this could explain the origin of the power-law scaling in the local field potential^55^. In the Bidomain model in the current study, a quasi-static assumption is used to derive the equations solely in terms of the electric potential of the intra and extracellular electric fields, but this assumption can only be made if the medium is frequency independent.

The neuronal model by Cressman et al.^6,7^ is developed to model the activity of one neuron. The model has been further developed in Wei et al.^56^ to incorporate chloride ion and oxygen concentrations. This extended model exhibit a broader range of neuronal activities such as seizures caused by hypoxia or cortical spreading depression. In contrast, an EEG is measured with electrodes placed on the scalp and represents an average of electrical activity within the brain. As such, the electric potential at a specific location of the skull is generated by the activity of neurons numbering in the tens of millions^49^. Our results here indicate that the Bidomain model has the potential to bridge the gap between these two scales in certain applications.

## Conclusion

This study shows that the Bidomain model gives rise to plausible power-spectra in the extracellular potential in idealised and realistic models of the brain. Neither an anisotropic conductivity tensor, nor varying the thickness of the CSF, skull, or scalp had any discernible effect on power-law distribution in the PSDs in any of the models. The WM cell model has a profound effect on the power spectra in the 2D models, but not in the three-dimensional models, in the current study.

## Data Availability

Meshes used on this study are available from the corresponding author upon request.
